# Human Health Applications of Calcium Montmorillonite Clay: A Systems-Based Review

**DOI:** 10.7759/cureus.95449

**Published:** 2025-10-26

**Authors:** Mitchell K Ng, David J Jacofsky, Wael K Barsoum, Michael A Mont

**Affiliations:** 1 Orthopedic Surgery, Rothman Orthopedic Institute, Philadelphia, USA; 2 Orthopedic Surgery, The CORE Institute, Phoenix, USA; 3 Orthopedic Surgery, Healthcare Outcomes Performance Company, Phoenix, USA; 4 Orthopedic Surgery, LifeBridge Health, Baltimore, USA

**Keywords:** calcium montmorillonite, clay, clinical applications, gastroenterology, inflammatory mediator

## Abstract

Calcium montmorillonite (CMM) clay is a naturally occurring mineral with a longstanding history in medical applications, now receiving increased scientific attention for its broad therapeutic potential. Known for its ability to bind a range of harmful substances, including bacterial toxins, heavy metals, mycotoxins, and inflammatory mediators, it exerts its effects without systemic absorption, acting locally within the gastrointestinal tract or at the skin surface. This narrative review synthesizes current clinical and preclinical evidence on the human health applications of calcium montmorillonite, with focused sections on the gastrointestinal/metabolic, dermatologic, immune, and musculoskeletal systems. The gastrointestinal section covers its documented role in treating pediatric diarrhea, radiation enteritis, and dietary toxin exposure. In dermatology, the clay has been incorporated into topical preparations for acne, rashes, and wound care, supported by both laboratory data and real-world use. Hepatic and metabolic studies suggest that it may reduce liver fat accumulation, improve glucose metabolism, and modulate the gut microbiome, particularly in models of non-alcoholic fatty liver disease and obesity. Additional sections explore its potential relevance in renal toxin clearance, immune regulation, mucosal healing, and surgical recovery. Across these systems, calcium montmorillonite has demonstrated a strong safety profile, with minimal nutrient interaction and no evidence of systemic toxicity when properly sourced and used in appropriate contexts. With growing access to carefully studied, pharmaceutical-grade formulations, CMM may offer safe and consistent benefits across clinical and preventive care settings.

## Introduction and background

Calcium montmorillonite (CMM) is a naturally occurring smectite clay formed from the weathering of volcanic ash, characterized by a high surface area, layered silicate structure, and exchangeable calcium ions [1]. Although clays have been employed therapeutically for millennia, calcium montmorillonite has emerged as a distinct material of interest due to its physicochemical stability and broad toxin-binding capabilities [2]. Historical records from ancient Egyptian, Mesopotamian, and various indigenous medical traditions describe the use of natural earth materials for the treatment of gastrointestinal illness, skin infections, and environmental poisonings [1,3]. While the specific identification of calcium montmorillonite as separate from other clays is relatively recent, its use in traditional medicine reflects longstanding empirical recognition of its safety and efficacy in both oral and topical contexts [3-5].

Calcium montmorillonite exhibits lower swelling capacity in aqueous environments, but greater structural stability and superior-binding affinity for a range of organic and inorganic toxins [1]. These include bacterial endotoxins, mycotoxins, pesticides, and heavy metals. Its mechanism of action is localized, functioning primarily within the gastrointestinal tract or on epithelial surfaces, without systemic absorption [6]. This property allows it to sequester harmful substances while minimizing adverse systemic effects. Clinical and experimental evidence support its use in the management of diarrhea, aflatoxin exposure, radiation enteritis, and inflammatory dermatologic conditions [5,7-9].

In addition to its adsorptive properties, calcium montmorillonite exhibits anti-microbial activity. In vitro studies have demonstrated inhibition of several clinically relevant bacterial species, including *Escherichia coli*, *Staphylococcus aureus*, and *Pseudomonas aeruginosa* [6]. Proposed mechanisms include disruption of bacterial membranes through surface interactions and interference with microbial metabolism via cation exchange [1]. These effects have led to the incorporation of calcium montmorillonite into wound dressings and topical formulations [1]. Furthermore, recent investigations have explored its utility in musculoskeletal applications. Its biocompatibility and structural characteristics make it a candidate for use in bone regeneration, localized drug delivery systems, and orthopedic biomaterials [10-12]. Preliminary data suggest it may enhance osteoblastic activity and support bone healing when used alone or in combination with therapeutic agents [10].

This narrative review provides a comprehensive evaluation of calcium montmorillonite’s biomedical applications, organized by organ system, including (1) gastrointestinal/metabolic, (2) dermatologic, (3) immune/inflammatory, and (4) perioperative, with a focus on musculoskeletal systems. By integrating findings from historical use, preclinical models, and emerging clinical studies, this review aimed to comprehensively characterize the clinical potential of calcium montmorillonite and identify directions for future translational research.

## Review

Overview of clinical applications

Calcium montmorillonite is a non-absorbable smectite clay with a high-binding capacity for bacterial toxins, mycotoxins, bile acids, heavy metals, and other inflammatory compounds [1]. Its mechanism of action is localized to epithelial surfaces, where it adsorbs harmful substances without entering systemic circulation [6]. This property, combined with its chemical stability and low toxicity, makes it suitable for a variety of therapeutic applications. Clinical and preclinical studies have most extensively examined its use in gastrointestinal and dermatologic contexts, where it improves stool quality, reduces mucosal inflammation, supports skin healing, and inhibits microbial growth (Table 1) [7-10,13].

**Table 1:** Summary of clinical benefits of calcium montmorillonite.

Clinical domain	Reported benefits
Gastrointestinal/metabolic	Binds bacterial toxins, aflatoxins, and bile acids; improves stool consistency; reduces gut inflammation; supports mucosal barrier function; modulates gut microbiota; lowers circulating endotoxin levels; reduces hepatic steatosis in preclinical models; modulates bile acid metabolism and glucose regulation; may improve weight regulation in obesity models (preclinical).
Dermatologic	Reduces acne-related inflammation; absorbs exudate in wounds and dermatitis; enhances skin barrier repair; inhibits bacterial growth on skin; and provides trace minerals that may support hair and scalp health.
Immune and inflammatory	Attenuates proinflammatory cytokines; reduces low-grade systemic inflammation; may influence gut-immune signaling.
Perioperative and musculoskeletal	Supports wound healing postoperatively; enhances osteoblast activity; functions as scaffold material in bone regeneration; potential vehicle for local antibiotic delivery.

Emerging evidence suggests broader physiological effects through gut-mediated pathways. By reducing intestinal permeability and circulating endotoxin levels, calcium montmorillonite may influence metabolic regulation, hepatic inflammation, and immune signaling [2,9]. In animal models, it has been associated with improved insulin sensitivity, reduced hepatic steatosis, and attenuation of systemic inflammation [3,9]. These effects extend its relevance to conditions, such as metabolic syndrome, non-alcoholic fatty liver disease, and possibly immune-mediated diseases. In addition, there is growing interest in its potential to influence respiratory health via the gut-lung axis, with historical use supporting its role in managing chest inflammation and airway irritation [14].

In surgical and musculoskeletal settings, calcium montmorillonite has been studied as a wound dressing component and a scaffold for bone regeneration [10,11]. Its porous structure allows for the delivery of local therapeutics and supports osteoblast activity in preclinical models [15]. As therapeutic applications expand, attention to pharmaceutical-grade sourcing and quality control remains essential [16]. Properly prepared calcium montmorillonite formulations must meet safety standards for purity, particle size, and absence of heavy metals or microbial contamination, ensuring consistent performance across clinical settings.

Gastrointestinal/metabolic

Calcium montmorillonite (CMM) has long been utilized in the treatment of gastrointestinal disturbances, owing to its strong adsorptive properties and favorable safety profile (Table 2). As a non-absorbed, locally acting agent, CMM binds a wide range of enteric toxins, bile acids, and microbial byproducts, forming inert complexes that are excreted via the feces [17,18]. This mechanism underlies its traditional and modern use in treating acute diarrheal illnesses. In pediatric populations, montmorillonite-based compounds remain a mainstay of care for non-specific diarrheal syndromes in various global health settings. A multicenter clinical study of 50 patients conducted in China demonstrated that co-administration of CMM with zinc sulfate and vitamin A/D supplements significantly enhanced treatment efficacy, resulting in faster resolution of symptoms and marked reductions in inflammatory biomarkers, including tumor necrosis factor (TNF)-α, nitric oxide, and C-reactive protein [17].

**Table 2:** Clinical studies highlighting gastrointestinal effects of montmorillonite. IFN-γ: interferon gamma; n: number of patients

Studies	Year	Study type (n)	Findings
Wang et al. [17]	2021	Prospective (156)	Children with diarrheal diseases receiving montmorillonite, vitamin A/D, and zinc for seven days had decreased inflammatory factor levels, time to onset of symptom relief, and improved T-lymphocyte levels (p<0.05).
Chen et al. [19]	2021	Prospective (43)	Montmorillonite powder with dexamethasone was associated with decreased levels of IL-2 and IFN-γ (p<0.001), decreased mucosal damage, and overall curative effect (p<0.05).
Awuor et al. [20]	2016	Prospective (50)	Administration of calcium montmorillonite clay led to decreased levels of aflatoxin B1-lysine in both serum and urine (p<0.05, β=0.49, 95% confidence interval: 0.32-0.75).
Wang et al. [9]	2005	Prospective (50)	Calcium montmorillonite clay associated with no significant adverse effects, no differences in hematology, liver/kidney function, electrolytes, and minerals in either group.
Ducrotte et al. [21]	2005	Prospective (524)	Patients with diarrhea-predominant irritable bowel syndrome who received montmorillonite experienced more improvement in abdominal pain, less discomfort, and higher overall success (p<0.016).
Guarino et al. [22]	2001	Prospective (804)	Administration of smectite to children with acute diarrhea was associated with reduction of duration of diarrhea, stool frequency, and consistency (p<0.01).

CMM has also shown benefit in mitigating mucosal injury associated with radiation therapy. In a randomized trial involving 86 patients with radiation-induced enteritis, rectal administration of CMM combined with dexamethasone led to significantly improved endoscopic and histopathologic scores (p<0.05), along with reductions in circulating interleukin (IL)-2 and interferon (IFN)-γ levels (p<0.001) [19]. These findings suggest a broader role for CMM in conditions involving epithelial barrier disruption and cytokine-mediated inflammation [19].

Beyond these applications, there is growing interest in the use of montmorillonite clays as modulators of host-microbiome interactions. A study by Xu et al. using a high-fat diet mouse model demonstrated that CMM significantly reduced luminal free fatty acids and endotoxin concentrations, in part by physically binding these molecules in the gut. This effect was associated with favorable shifts in the gut microbial community, specifically increased abundance of Blautia (a genus of short-chain fatty acid producers) and a reduction in Desulfovibrio (a prominent lipopolysaccharide {LPS}-producing genus) [6]. These microbial and metabolic changes correlated with improvements in glucose tolerance and reduced hepatic steatosis, implicating a role for CMM in modifying the gut-liver axis. In addition, a randomized clinical trial of 50 patients found that calcium montmorillonite dietary inclusion led to decreased aflatoxin concentrations in both serum and blood [20]. There is also interest in the use of CMM on mineral nutrition and skeletal health. The calcium content of the clay provides an accessible source of this essential mineral, which aligns with experimental findings of improved bone density and mineralization [21,22]. In addition, some naturally occurring forms, such as red clay, carry a broad profile of more than 50 trace elements (e.g., zinc, copper, magnesium, and manganese) that are readily absorbed and play key roles in collagen cross-linking, enzymatic activity, and tissue repair [14,19]. This mineral richness may underlie part of the benefit observed in metabolic and musculoskeletal studies, though clinical trials will be required to confirm these effects in patients [15]. Together, these findings position calcium montmorillonite as more than a symptomatic agent. Its capacity to sequester bioactive lipids and microbial products, reinforce mucosal integrity, and remodel the intestinal microbiota suggests therapeutic potential across a range of gastrointestinal disorders - from acute enteritis to microbiome-linked metabolic dysfunctions.

Dermatology

Montmorillonite-based clays, including quaternium-18 bentonite, have been clinically evaluated for their effectiveness in managing several common skin conditions, particularly those involving barrier disruption and irritant exposure [23]. These clays form a physical film on the skin surface, adsorbing irritants and allergens while reducing transdermal absorption of inflammatory compounds.

In a clinical trial involving 37 patients with a known history of chronic hand dermatitis - either allergic or irritant in nature - those who were administered a skin moisturizing cream containing quaternium-18 bentonite showed a 50% improvement in skin condition (p<0.001) and decreased corticosteroid usage [24]. Although this study lacked a control group, the high response rate supports its use as an adjunct in barrier-restoring regimens. A subsequent randomized multicenter clinical trial of 211 subjects with allergic contact dermatitis revealed that patients pretreated with quaternium-18 bentonite lotion had absent or significantly reduced reactions to urushiol relative to untreated sites (p<0.0001) [25]. Apart from protection against dermatitis, a clinical trial of 75 subjects with oily/acne skin who used a bentonite clay mask had significant improvements in acne, sebum content, stratum corneum water content, and transepidermal water loss [26]. Combined, these studies highlight the clay’s role as an effective barrier against cutaneous allergen penetration and excessive sebum production.

In pediatric dermatology, diaper dermatitis remains one of the most common inflammatory conditions of infancy. A double-blind randomized clinical trial (RCT) of 100 infants with infantile diaper dermatitis found that bentonite-based cream resolved skin symptoms more quickly and effectively than Calendula ointment, a widely used botanical treatment [27]. By hour 6, 88% of infants treated with bentonite showed symptom resolution compared to 54% in the Calendula group (p<0.001). A separate double-blind RCT of 60 outpatient infants with diaper dermatitis found that patients treated with bentonite instead of Calendula had improved rates of recovery at both 6 hours (93 versus 40%, p<0.001) and three days (90 versus 37%, p<0.001) [28]. Taken together, these findings reinforce the rapid soothing and protective effect of bentonite in pediatric skin conditions. Although large-scale trials are limited, available and growing clinical evidence supports the safe and effective use of montmorillonite-derived products in various dermatologic contexts. Their favorable safety profile, lack of systemic absorption, and compatibility with both adult and pediatric skin make them appealing candidates for continued therapeutic use in barrier-related dermatoses (Table 3).

**Table 3:** Clinical studies highlighting dermatologic effects of montmorillonite. n: number of patients

Studies	Year	Study type (n)	Findings
Zhang et al. [26]	2023	Prospective (75)	Patients with oily/acne skin who utilized clay mask had improvements in acne-related outcomes, sebum content, skin evenness, stratum corneum water content, and transepidermal water loss.
Mahmoudi et al. [27]	2015	Prospective (100)	Patients with infantile diaper dermatitis who received bentonite had improvement in the first 6 hours relative to those who received Calendula (88 versus 54% (p<0.001).
Adib-Hajbaghery et al. [28]	2014	Prospective (60)	Patients with diaper dermatitis who received bentonite cream experienced improved recovery relative to Calendula at both 6 hours (93 versus 40%, p<0.001) and three days (p<0.001).
Fowler Jr [24]	2001	Prospective (37)	Patients with chronic hand dermatitis were administered cream containing quaternium-18 bentonite, decreasing corticosteroid usage with skin improvement of 50% (p<0.001).
Marks et al. [25]	1995	Prospective (211)	Patients with a history of allergic contact dermatitis to poison ivy and poison oak who were pretreated with bentonite lotion had absent/reduced reactions to urushiol relative to control sites (p<0.0001) on all test days.

Immune/inflammatory

Though not designed to measure cytokines or inflammatory mediators, the favorable outcomes in these patients with inflammatory dermatologic conditions like hand/diaper dermatitis further support the hypothesis that montmorillonite may reduce inflammatory signaling. To this end, although traditionally used for its physical adsorption and barrier-forming properties, calcium montmorillonite (CMM) clay is increasingly recognized for its potential anti-inflammatory effects, both at mucosal surfaces and systemically. These effects appear to arise not through direct pharmacologic action, but via indirect mechanisms, such as toxin sequestration, microbiota modulation, and epithelial barrier stabilization.

In an in vivo poultry model, Wang et al. demonstrated that copper-loaded modified montmorillonite (MMT) improved growth performance in broilers by attenuating intestinal inflammation and enhancing immune homeostasis [29]. MMT administration was associated with significantly downregulated pro-inflammatory cytokines (IL-1β, IL-6, tumor necrosis factor-alpha {TNF-α}), reduced serum myeloperoxidase (MPO) and nitric oxide (NO) levels (p<0.05), and upregulated anti-inflammatory cytokines, such as IL-4 and IL-10 (p<0.05). These effects were mediated via inhibition of the TLRs/MAPK/NF-κB signaling pathway and were accompanied by reduced expression of key upstream regulators (TLR2, TLR4, Myd88, TRAF6) and inflammatory effector proteins (iNOS, NF-κB, p38, ERK) [29]. Notably, MMT also enhanced the expression of intestinal tight junction proteins (claudin-1, occludin, ZO-1), further supporting its protective effect on the mucosal barrier.

A randomized controlled trial of 156 pediatric patients with acute diarrheal disease compared standard CMM therapy to a combination of CMM with zinc sulfate and vitamins A/D [17]. The combination group showed significantly lower serum levels of pro-inflammatory markers, including tumor necrosis factor-alpha (TNF-α), nitric oxide (NO), C-reactive protein (CRP), and procalcitonin (PCT) after seven days of treatment (p<0.05). These reductions were accompanied by shorter durations of hospitalization and symptom resolution, suggesting that local toxin sequestration by CMM may help dampen systemic inflammatory cascades [17].

Although additional clinical studies are needed to directly quantify cytokine responses in non-GI inflammatory conditions, current evidence supports the hypothesis that calcium montmorillonite can reduce systemic inflammatory signaling by mitigating exposure to gut-derived toxins. Quantitative analysis after application of clay minerals, sepiolite, and palygorskite revealed mRNA expression of inhibition of a host of cytokines, including IL-1, IL-6, tumor necrosis factor (TNF-α), and interferon gamma (IFN-γ), with decreased polymorphonuclear peroxidase activity [30]. Taken together, the available evidence suggests that CMM may serve as a supportive adjunct in conditions marked by mucosal immune activation and persistent low-grade inflammation, with potential relevance not only to autoimmune disorders but also to gastrointestinal allergy states where barrier dysfunction is a central feature.

Perioperative

Although calcium montmorillonite has not yet been adopted in standard surgical protocols, its distinctive chemical and biological properties suggest considerable promise in the perioperative setting [1]. Its ability to bind endotoxins, stabilize epithelial surfaces, and reduce pro-inflammatory signaling makes it a potential asset in managing common complications, such as postoperative ileus, low-grade systemic inflammation, and delayed tissue healing [9,31].

In orthopedic contexts, CMM has demonstrated particular utility when combined with calcium phosphate cements (CPCs) to enhance their mechanical properties (Table 4). A study by Wei et al. evaluated CPCs reinforced with varying concentrations of CMM [15]. At 50% CMM incorporation, the compressive strength increased to 48.5 MPa, representing a 227% improvement compared to CPC alone (14.83 MPa). The elastic modulus increased modestly from 1.53 to 1.85 GPa, indicating enhanced load-bearing capability. Of note, the anti-washout properties were also substantially improved, with mass loss in solution reduced from 71.25% to just 6.48%, making the material more stable during surgical handling and early implantation [15]. A separate study of montmorillonite clay coating titanium implants was associated with enhanced local osseointegration and angiogenesis in vitro [32].

**Table 4:** Basic science studies highlighting perioperative orthopedic effects of montmorillonite.

Studies	Year	Study type	Findings
Xiong et al. [11]	2024	Basic science	Montmorillonite clay coating was associated with increased osseointegration and angiogenesis of titanium implants.
Wei et al. [15]	2022	Basic science	Composite calcium phosphate/montmorillonite demonstrated enhanced compressive strength (227%) and anti-washout ability relative to normal cement.
Kim et al. [10]	2020	Basic science	Nano-montmorillonite demonstrated osteogenic effects, improving alkaline phosphatase activity, expression of osteoblast differentiation genes, and inhibiting cathepsin K and nuclear factor kappa-B ligand.

In addition, there is growing evidence that CMM has the potential for positive systemic effects on bone health. The application of nano-montmorillonite scaffold significantly increased osteogenic expression, including a 2.5-fold increase in RUNX2 and 2.2-fold increase in osteocalcin relative to controls [10]. The novel montmorillonite scaffold also reduced TNF-α and IL-6 expression by approximately 40% and 35%, respectively, in LPS-stimulated RAW264.7 macrophages, indicating anti-inflammatory potential [10]. The overall effect was an enhancement of osteogenic differentiation, increased expression of osteoblast genes, alkaline phosphatase activity, and mineralization in both an osteoblastic cell line in vitro and an osteoporosis model in vivo [10].

Future directions

Ongoing research into calcium montmorillonite should prioritize large-scale clinical trials to better define its therapeutic role across a range of conditions. While current evidence supports its use in toxin-related gastrointestinal disorders and dermatologic applications, more data are needed to evaluate its long-term impact in chronic diseases, such as irritable bowel syndrome, inflammatory bowel disease, non-alcoholic fatty liver disease, and metabolic dysfunction. Mechanistic studies should further investigate its influence on intestinal barrier integrity, mucosal immune responses, and gut-derived systemic inflammation. Because of its effects on gut barrier function and the microbiome, CMM may have relevance in metabolic conditions, such as obesity, where low-grade inflammation and altered nutrient handling play central roles. Early observations have also raised the question of whether trace mineral content or anti-inflammatory activity might contribute to improvements in disorders like hair loss, though this remains to be tested formally. Likewise, the ability of CMM to reduce mucosal immune activation suggests a potential role in autoimmune disease and in gastrointestinally mediated allergic reactions.

At a translational level, greater attention is needed to optimize pharmaceutical-grade clay formulations, ensuring consistent particle size, mineral composition, and absence of heavy metals or microbial contaminants. Its structural properties make it a promising platform for localized drug delivery or incorporation into wound dressings and bone scaffolds. Additional studies should also evaluate its use in vulnerable populations, such as children and older adults, as well as compare its efficacy to other adsorptive agents in both acute and chronic settings. Finally, collaboration with regulatory agencies will be important to establish international standards for clinical use, enabling broader and safer access to high-quality calcium montmorillonite products, particularly in toxin-endemic regions.

An important step in moving CMM into routine clinical use will be to define dosing strategies that are both effective and practical. Many commercial preparations provide little direction on therapeutic amounts, which may explain some of the inconsistency in reported outcomes. A modified activation process increases the surface area and binding activity of CMM+, allowing therapeutic effects to be achieved with smaller quantities. In practice, this translates to roughly one teaspoon per day, a considerably lower dose than is typical with conventional clays. This distinction not only supports patient adherence but also highlights the value of standardized, well-characterized formulations in realizing the clinical potential of CMM.

## Conclusions

Calcium montmorillonite is a naturally-derived compound with a unique combination of toxin adsorption, barrier stabilization, and immune modulation, offering significant promise across multiple domains of human health. Its clinical utility is supported by a growing body of both basic science and clinical evidence in gastrointestinal, dermatologic, inflammatory, and perioperative settings, with benefits ranging from local detoxification to enhanced wound healing and osteogenesis. Given its favorable safety profile and broad applicability, calcium montmorillonite has significant potential as an adjunctive therapy that merits further translational research and possible routine clinical integration. Further studies should aim to more fully characterize its benefits and identify the patient cohorts for which it is best indicated.
